# Re-Examination of Inflammation in Major Depressive Disorder: Bridging Systemic and Neuroinflammatory Insights

**DOI:** 10.3390/biom15111556

**Published:** 2025-11-05

**Authors:** Xinyu Ye, Yuen-Shan Ho, Raymond Chuen-Chung Chang

**Affiliations:** 1 Laboratory of Neurodegenerative Diseases, School of Biomedical Sciences, LKS Faculty of Medicine, The University of Hong Kong, Pokfulam, Hong Kong SAR, China; 2 School of Nursing, Faculty of Health and Social Sciences, The Hong Kong Polytechnic University, Hung Hom, Hong Kong SAR, China

**Keywords:** major depressive disorder, neuroinflammation, systemic inflammation, systemic-CNS inflammation interaction, brain lymphatic system, heterogeneity of MDD

## Abstract

Major depressive disorder (MDD) is a multifaceted psychiatric disorder that has been a longstanding focus of research. However, its underlying mechanisms remain underexplored. Recently, the inflammatory hypothesis has gained attention, highlighting inflammation’s role in MDD progression. Potential contributors to increased systemic inflammation in MDD include hyperactivation of the hypothalamic–pituitary–adrenal axis, dysregulation of the sympathetic nervous system, gut microbiota imbalances, the “pathogen host defense” hypothesis, and damage-associated molecular patterns. Traditional pathways explaining how systemic inflammation affects the central nervous system (CNS) do not fully account for the observed desynchrony between systemic and neuroinflammation in most depressed individuals. Alternative models suggest mechanisms such as reduced blood–brain barrier permeability and the involvement of immune cells from the skull. This review examines the link between inflammation and MDD, focusing on systemic and neuroinflammation interactions, with special emphasis on the heterogeneity of MDD symptoms and the potential impact of dysfunction in the brain’s lymphatic system. Gaining insight into the origins of inflammation in both the central nervous system and the peripheral body, along with their interactions, offers an important understanding of the inflammatory mechanisms associated with MDD for future treatment.

## 1. Introduction

Major depressive disorder (MDD) is a complex and debilitating mental illness that has long been the focus of scientific research. It affects approximately 280 million people around the world, and twenty percent of people will experience depression in their lives [1]. Over the years, researchers have explored various factors that contribute to depression, including genetic predisposition, environmental stressors, and imbalances in neurotransmitter levels [2]. Currently, the majority of antidepressants prescribed for MDD patients primarily target the monoaminergic system, effectively increasing serotonin and noradrenaline levels. However, these medications are only effective for approximately two-thirds of MDD patients, indicating that additional mechanisms may exist [3]. Indeed, neuroplasticity theory, neurotrophic factor theory, and hypothalamic–pituitary–adrenal (HPA) axis theory have been proposed, highlighting the involvement of impaired structural plasticity, reduced neurotrophic factor concentration, and HPA axis hyperactivation in depression, respectively. Nevertheless, none of the current theories can comprehensively account for the complexity of depression.

In recent years, emerging research on human MDD patients and animals has shed light on novel connections between depression and inflammatory responses. Evidence comes from a combination of epidemiological studies that have identified a link between depression and elevated immune markers in the blood [4], as well as findings from both pre-clinical and clinical studies that demonstrate the generation of depressive symptoms after exposure to immune stimulation [5], indicating a bidirectional relationship between immune responses and depressive symptoms. Indeed, increased levels of pro-inflammatory cytokines have been found in the peripheral circulation and cerebrospinal fluid of MDD patients, alongside the activation of immune cells in both the peripheral system and the brain [6]. A survey conducted on COVID survivors revealed a positive association between the systemic inflammation index and ratings of depression and anxiety [7]. Furthermore, an 18-year longitudinal study demonstrated that the severity of depressive and anxiety disorders predicted increased peripheral immune responses, which further forecasted worse executive function performance nine years later [8]. Nevertheless, findings from various studies have been inconsistent, and the sources of this inflammation, as well as the relationship between peripheral inflammation and neuroinflammation observed in MDD patients, are still not fully understood.

Extensive research and review have been conducted to investigate the sources of inflammation in MDD patients, focusing particularly on the HPA axis, the sympathetic nervous system, gut microbiota, and oxidative stress [9,10]. The presence of pro-inflammatory cytokines and acute phase proteins in the peripheral bloodstream may indicate neuroinflammation, as these cytokines generated in the peripheral tissues are capable of traversing the BBB. Once entering the brain, they affect stress response systems, adult neurogenesis, and neural plasticity, leading to the development of depressive symptoms [9,11]. As a result, many studies highlight the interplay between systemic and neuroimmune responses in patients with MDD, emphasizing the involvement of systemically derived cytokines and immune cells infiltrating the central nervous system. However, it is essential to recognize that most human MDD studies have not found a direct correlation between systemic inflammation and neuroinflammation in patients, challenging the traditional view that circulating cytokines and leukocytes enter the brain and contribute to neuroinflammation and depressive symptoms [6]. Moreover, the correlations between inflammatory markers and MDD appear to vary depending on the symptom type, with the strongest associations observed in somatic symptoms rather than cognitive ones [12].

The evolving understanding of the relationship between depression and inflammatory responses opens new avenues for exploring innovative treatments and has the potential to change the way we perceive and manage this prevalent mental health condition. In this review, we thoroughly analyze the available evidence from both human and animal studies on depression, focusing specifically on the relationship between peripheral inflammation, neuroinflammation, and depressive symptoms.

## 2. Inflammatory Responses in Human MDD Patients

### 2.1. Systemic Immune Responses in MDD Patients

**Figure 1:**
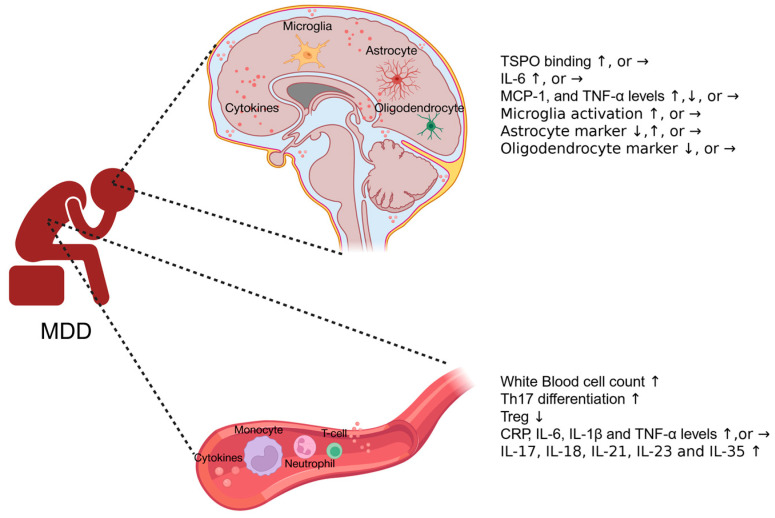
Systemic and neuroinflammation in MDD patients. In general, MDD patients exhibit elevated inflammatory responses in both the central nervous system and the bloodstream, as indicated by increased white blood cell counts, higher levels of pro-inflammatory cytokines, and activated microglia, although some confounding results have been reported. ↑: increase; ↓: decrease; →: no significant change.

Clinical depression has been associated with various inflammatory conditions or disorders, such as obesity, autoimmune diseases, as well as the postpartum period. Mounting evidence support a correlation between depression and increased levels of immune markers in the peripheral blood and in the central nervous system (Figure 1). Table 1 summarizes the changes in circulating inflammatory markers in patients with MDD. A comprehensive meta-analysis encompassing 27 studies reveals that the average white blood cell count is significantly elevated in individuals with MDD compared to control groups, suggesting a modification in the composition of peripheral blood cells in MDD patients [13]. Monocytes are innate immune cells involved in both the initiation and regulation of immune responses. Based on the surface antigen expression, circulating monocytes can be classified into three subgroups: CD16^neg^CD14^++^ classical monocytes, CD16^+^CD14^+^ intermediate monocytes, and CD16^++^CD14^neg^ non-classical monocytes [14]. A cross-sectional study on MDD patients found a positive correlation between higher levels of intermediate and non-classical pro-inflammatory monocytes and the severity of depression, while higher levels of the total count of monocytes appear to have a protective effect [15]. This highlights the complex interplay between different immune cell types and the disease severity of MDD.

Within the broad range of serologic indicators for systemic inflammation, interleukin-6 (IL-6), which stimulates the production of C-reactive protein (CRP), has received significant attention in extensive research [16]. As a pro-inflammatory cytokine, increased levels of IL-6 have been shown to affect inhibitory synapse function and excitation-inhibition balance, leading to cognitive dysfunction and mood disorders [17]. MDD patients exhibit significantly elevated levels of CRP, IL-6, and Tumor necrosis factor-alpha (TNF-α) in their plasma compared to healthy individuals, which significantly decreased after 8 weeks of SSRI treatment [18,19]. Moreover, the expression levels of IL-17, IL-18, IL-21, IL-23, and IL-35 were substantially elevated in patients with depression [20,21,22]. In contrast, the mRNA expression of Foxp3 was reduced in patients with MDD, consistent with previous results suggesting decreased T regulatory cell levels in depressed patients [21,23]. The largest case study involving approximately 27,000 MDD patients from the UK Biobank demonstrated a significant association between elevated blood levels of CRP and depression [24]. In addition, a subset of depressed individuals who are less likely to experience positive outcomes from conventional antidepressant treatments (nortriptyline and escitalopram) are consistently distinguished by heightened baseline levels of IL-1β, MIF, and TNF-α [25]. Moreover, the resolution of depression symptoms is generally followed by the restoration of normal levels of immune markers [26].

However, such an association between inflammation and depression is not always consistent, varying across studies and cytokines. For example, some studies found no significant difference in plasma Interleukin-1β (IL-1β), TNF-α, or IL-6 levels between MDD and healthy controls, nor between pre- and post-treatment [27,28]. Melancholic patients of acute MDD showed no change in the inflammation profile, while non-melancholic patients exhibited significantly higher levels of IL-1β. Young adults who have experienced childhood trauma exhibit heightened inflammation, evident through increased levels of CRP and the count of white blood cells, regardless of their depression diagnosis [29].

**Table 1:** Change in inflammatory markers in the peripheral blood of MDD patients.

Author	Subject Condition	Inflammatory Marker	Results
Foley et al. [13]	Meta-analysis: depression diagnosed clinically	Peripheral blood composition.	Increased counts of total white blood cells, monocytes, neutrophils, granulocytes, natural killer cells, CD19+ B cells, and CD4+ T helper cells. Decreased percentage of lymphocytes.
Alvaarez-Mon et al. [22]	Psychiatrist-confirmed MDD with a minimum score of 14 points on the Hamilton Rating Scale for Depression, aged between 18 and 65.	Peripheral CD4+ T lymphocyte subset distribution, activation, and differentiation states	Increased serum levels of IL-17 and TNF-α, and increased Th17 differentiation in circulating CD4+ T cells of MDD patients.
Li et al. [23]	Clinically diagnosed with MDD for the first time, and without previous use of antidepressants.	T helper cell cytokine and CD4+CD25+ T regulatory cell levels	Increased Th1/Th2 ratio and decreased CD4+CD25+ T regulatory cell levels.
Tannous et al. [18]	Volunteers aged between 18 and 65 meeting the DSM-IV diagnosis of MDD.	CRP, IL-6 levels in the blood	Significantly higher IL-6 levels and a trend for increased CRP levels.
Pitharouli et al. [24]	Diagnosed with MDD from the Composite International Diagnostic Interview	Serum CRP level	Significantly higher levels of CRP. Depression is significantly associated with increased Log CRP levels.
Gattaneo et al. [25]	Patients aged between 19 and 72, suffering from moderate to severe unipolar depression.	IL-1α, IL-1β, IL-4, IL-6, IL-7, IL-8, IL-10, MIF, and TNF-α levels in the blood.	Higher mRNA levels of IL-1β, IL-6, MIF, and TNF-α. Lower levels of IL-4. No significant change in IL-1α, IL-7, IL-8, and IL-10. Antidepressant non-responders have higher baseline mRNA levels of IL-1β, MIF, and TNF-α.
Yin et al. [19]	Patients aged between 18 and 72, and first-time diagnosed with depression according to ICD-10.	IL-1, IL-6, IL-10, TNF-α, and hs-CRP in the peripheral blood.	Significantly higher levels of IL-1, IL-6, IL-10, TNF-α, and hs-CRP.
Kobayashi et al. [30]	Diagnosed with MDD using DSM-IV criteria by psychiatrists.	Blood mRNA levels of IL-1β, IL-6, TNF-α, IL-10, IL-1RA, SOCS1, SOCS2, and SOCS3. Blood protein levels of IL-1β and IL-6.	mRNA levels of SOCS1, SOCS2 and SOCS3 are decreased. No increase in IL-1β, IL-6, TNF-α, IL-10, or IL-1RA.
Galecka et al. [21]	Patients aged between 20 and 67 and diagnosed with MDD according to ICD-10.	Serum protein and RNA levels of IL-17, IL-21, IL-23, IL-35, and Foxp3.	Gene expression levels of IL-17, IL-21, IL-23, and IL-35 are significantly increased.
Merendino et al. [20]	Female patients suffering from moderate-severe depression.	Serum IL-18 and CD30.	Significantly higher IL-18 levels and no change in CD30 levels.

### 2.2. Neuroimmune Responses in MDD Patients

#### 2.2.1. Neuroimaging and Cerebrospinal Fluid (CSF) Cytokine Profile in MDD Patients

Compared to investigations on periphery immune response in MDD patients, studies examining brain immune response are relatively limited. Existing investigations predominantly utilize positron emission tomography (PET), postmortem brain analyses, and the assessment of CSF immune markers. Similar to systemic immune responses, neuroinflammation is not always observed in MDD patients, with conflicting results depending on the approaches and markers being examined (Table 2).

Most studies investigating immune markers in the CSF of MDD patients focused on the expression levels of IL-6, IL-1β, and TNF-α but presented inconsistent results. A meta-analysis of studies on CSF cytokine profiles revealed significant elevations in IL-6 and TNF-α levels among patients in comparison to control groups. However, it should be noted that only the results pertaining to IL-6 remained significant when sensitivity analyses were conducted [6]. Meanwhile, another study successfully found a significant upregulation of IL-6 levels in the CSF of patients who attempted to commit suicide, with the elevation being more than 8-fold and positively correlated with the severity of the symptoms [31].

Microglia are specialized immune cells that serve as resident macrophages in the central nervous system (CNS) and play a crucial role in maintaining the health and functions of the brain. To date, activation of microglia has emerged as a highly promising indicator of neuroinflammation, leading numerous neuroimaging and post-mortem studies to primarily examine the activation states of microglia in patients with MDD. Translocator protein (TSPO) is an 18 kDa protein located in the outer mitochondrial membrane that is closely linked to the activation of microglia [32]. In terms of in vivo imaging on MDD patients, collective evidence suggests that patients exhibit increased TSPO binding compared to healthy subjects [33], although negative results were also found [34]. This variability in TSPO imaging results may be attributed in part to the choice of various binding ligands, each of which exhibits different binding specificity and sensitivity characteristics [35]. Moreover, results obtained from PET scanning should be interpreted with caution, as the implications of increased ligand binding remain controversial. An elevation in TSPO binding does not exclusively indicate the level of microglia activation; rather, it also reflects contributions from macrophages, endothelial cells, and astrocytes [36].

#### 2.2.2. Cytokine Expression and Glia Activation in Postmortem Brains

In line with the findings from CSF analyses and PET scans, examinations of cytokines, chemokines, and other immune markers in post-mortem brains of individuals with depression have yielded mixed results. Some studies found higher expression levels of Monocyte chemoattractant protein-1 (MCP-1) and TNF-α [37,38], some found reduced expression levels [39,40], and others did not detect a significant difference [41]. The failure to detect significant differences in pro-inflammatory markers between MDD patients and healthy controls may be due to low sensitivity of the test reagents, heterogeneity in the severity of depressive symptoms, and small sample size. In addition, confounding factors such as age, body weight, exercise frequency, and gender may also hinder the difference between individuals with MDD and healthy control groups.

Meta-analysis showed that among the eight studies that were conducted, the majority did not observe changes in microglia associated with depression [6]. Conversely, three studies suggested an increase in microglia density in depressed patients [42]. Such inconsistency may be due to multiple factors such as study design, brain region being examined, and disease stage. For example, neuroinflammation has been consistently observed in suicide victims who suffer from MDD or other types of mental illness, suggesting a strong association between suicide attempts and neuroinflammation [43]. Meta-analysis of studies investigating changes in astrocyte and oligodendrocyte markers in post-mortem brains of individuals with MDD also revealed inconsistent findings. Of the 26 studies analyzed, 13 reported decreased astrocyte marker expression in MDD patients, 2 indicated increased expression, and 11 failed to find a significant difference between MDD and control groups [6]. Furthermore, among the 6 studies that assessed oligodendrocyte markers, 4 demonstrated decreased activity in the prefrontal cortex of MDD patients [6].

**Table 2:** Change in inflammatory markers in the central nervous system of MDD patients.

Author	Tissue Type	Inflammatory Marker	Results
Holmes et al. [33]	PET study	TSPO	TSPO availability significantly increased in the anterior cingulate cortex.
Setiawan et al. [42]	PET study	TSPO	TSPO distribution volume significantly increased during major depressive episodes.
Hannestad et al. [34]	PET study	TSPO	No significant difference in TSPO levels.
Sasayama et al. [44]	CSF	IL-6	Significantly higher levels of IL-6.
Hestad et al. [45]	CSF	IL-6, TNF-α, and MCP-1	No significant difference in IL-6, TNF-α, or MCP-1 levels.
Torres-Platas et al. [37]	Post-mortem (dorsal anterior cingulate cortex)	MCP-1, IBA-1	Gene expression of IBA1 and MCP-1 significantly increased. IBA1-positive microglia density did not change, but increased proportion of primed microglia.
Dean et al. [38]	Post-mortem (frontal cortex)	Transmembrane TNF and soluble TNF	Transmembrane TNF is significantly increased, but no change in soluble TNF.
Clark et al. [39]	Post-mortem (ventrolateral prefrontal cortex)	IL-1β, IL-2, IL-4, IL-5, IL-6, IL-13, IL-33, IFN-γ, TNF-α, CCL2, COX2.	Significantly decreased levels of IL-33, IFN-γ, and TNF-α. Expression levels of other cytokines did not change.
Pantazatos et al. [40]	Post-mortem (dorsal lateral prefrontal cortex)	IL-8, MCP-1	Gene expression levels of IL-8 and MCP-1 significantly decreased.
Brisch et al. [46]	Post-mortem (dorsal raphe nucleus)	HLA-DR	No significant change in microglia density.
Cobb et al. [47]	Post-mortem (hippocampus)	GFAP	GFAP+ astrocyte density decreased in the left hippocampi.
Williams et al. [48]	Post-mortem (substantia nigra)	GFAP	No change in GFAP+ astrocyte.
Davis et al. [49]	Post-mortem (dorsolateral prefrontal cortex and the anterior cingulate cortex)	GFAP	Significantly increased GFAP reactivity in the dorsolateral prefrontal cortex layer I.
Hayashi et al. [50]	Post-mortem (frontopolar cortex)	Olig2	The number of olig2+ nuclei is significantly reduced.
Rajkowska et al. [51]	Post-mortem (white matter from the ventral prefrontal cortex)	CNPase	No significant change in the density of CNPase+ oligodendrocytes.

### 2.3. Inflammation and the Heterogeneity of MDD

Depression is characterized by a complex spectrum of affective states, encompassing more than one singular symptom, with its progression exhibiting considerable variability among individuals. Depressive symptoms are generally classified into two primary domains: cognitive-affective and somatic. The cognitive-affective domain encompasses indicators of emotional disturbances such as sadness and loneliness, whereas the somatic dimension pertains to more physical manifestations such as fatigue and low energy levels [12].

A significant limitation in the majority of MDD research is the reliance on a singular composite score for evaluating the disorder rather than considering its multifaceted components. For instance, a 26-week longitudinal study found no meaningful relationship between any of the systemic inflammatory markers and the overall depression score. However, several cytokines showed correlations with specific dimensions of depression, primarily the somatic “neurovegetative” symptoms dimension [52]. Research indicates that inflammatory markers and elevated cortisol levels are more closely linked to somatic aspects of MDD than to the cognitive-affective symptoms [53]. For example, even though correlations have been observed between IgM and IgA levels and MDD scores, the symptoms most closely aligned with these correlations pertain to “sickness behavior”, including fatigue and perceived infections [54]. Furthermore, depressive symptoms can manifest as either short-term episodic or long-term persistent forms; however, many studies overlook this distinction.

### 2.4. The Desynchrony of Systemic and Neuroinflammation in MDD Patients

**Figure 2:**
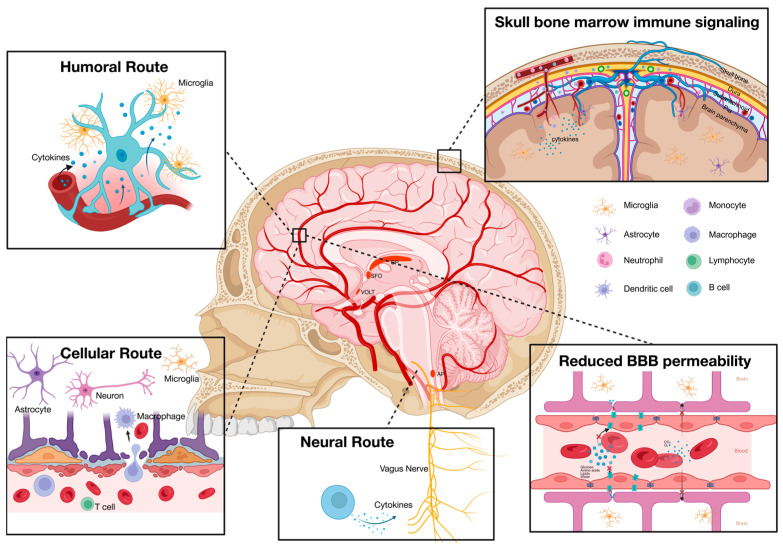
Schematic diagram illustrating possible pathways by which systemic and neuroimmune responses interact. Peripheral cytokines enter the central nervous system through the humoral route, where they traverse the blood–brain barrier via active transport or by crossing sites that lack a blood–brain barrier. These regions include the choroid plexus (CP), area postrema (AP), subfornical organ (SFO), and the vascular organ of lamina terminalis (VOLT). Circulating immune cells, such as monocytes, can penetrate the blood–brain barrier and access the central nervous system through the cellular route. In addition, peripheral immune signals can activate the vagus nerve and directly transmit signals to the central nervous system. There is bidirectional immune signaling between the brain parenchyma and the skull, making the skull a junction connecting peripheral immune responses and neuroinflammation. Moreover, systemic inflammation may reduce the permeability of the blood–brain barrier in some MDD patients, interfering with nutrient transport and neuronal activity, and ultimately leading to neuroinflammation. This may explain the desynchrony between peripheral immune signals and neuroimmune responses observed in MDD patients.

The brain has traditionally been regarded as an “immune-privileged” organ, characterized by a distinct separation from the body’s immune system. This concept originated in 1921 when Japanese researcher Shira observed that sarcomas could develop and persist within the CNS but failed to thrive when transplanted to the peripheral regions [55]. This “immune privilege” phenomenon has been primarily ascribed to the presence of the blood–brain barrier (BBB), which restricts the entry of peripheral molecules into the CNS. However, this notion has been challenged, suggesting that the brain’s “immune privilege” is relative rather than absolute. Figure 2 summarizes the possible pathways by which the systemic immune system and the neuroimmune system communicate.

It has been previously proposed that the systemic immune system communicates with the CNS through three primary routes: the neural route, the humoral route, and the cellular route. The neural route involves the generation of neural signals by peripheral inflammation. Such signals are transmitted to the brain via afferent nerves, functioning as part of the regulatory mechanisms for immune activity [56]. Meanwhile, systemic cytokines are able to access the brain via the humoral route through several mechanisms: active transport across the BBB, passage through the permeable regions of the circumventricular organs, or infiltration through a modulated BBB in pathological conditions. The entry of peripheral cytokines into the CNS triggers the activation of neuroimmune cells, including microglia, astrocytes, and oligodendrocytes, leading to an increase in cytokine production, oxidative stress, and neuronal dysfunction. In addition, peripheral immune cells are capable of migrating to the CNS through the cellular route, contributing to neuroinflammation, pathogen clearance, and tissue repair [57]. In studies using animal models of depression, long-term exposure to psychological stress significantly increased the migration of bone-marrow-derived monocytes to the hippocampus, where they differentiated into microglia. Furthermore, blocking the recruitment of these monocytes with C-C chemokine receptor antagonists effectively alleviated depressive-like behaviors [58]. Taken together, systemic immune mediators are proposed to be able to traverse into the CNS during inflammatory states, contributing to neuroinflammation and the development of cognitive and psychiatric disorders, such as depression [59].

However, this theory failed to explain the desynchrony observed among PET scan results, CSF cytokine levels, and plasma immune markers in patients with MDD (Table 3). Notably, most human studies examined neuroinflammation and systemic inflammation independently due to ethical restrictions and resources available. However, in those studies that examined systemic inflammation and neuroinflammation in MDD patients concurrently, elevated peripheral immune responses were not always accompanied by neuroinflammation. A meta-analysis examining the correlations between systemic and neuroinflammation indicates that among 9 PET and CSF studies conducted on individuals diagnosed with depression, 7 studies failed to identify a significant association between CNS inflammatory markers and systemic inflammatory markers [6]. Only 2 studies found a weak correlation between a few immune markers in CSF and serum of patients [45,60]. One study identified a significant and robust positive correlation between CRP levels in the CSF and plasma but not other cytokines or immune markers [61]. Moreover, no significant association was found between brain TSPO binding level, peripheral CRP concentration, or other peripheral inflammatory markers [33,62]. As a result, the relationship between systemic inflammation and neuroinflammation in MDD patients, as well as the mechanisms that account for the observed desynchrony, remain unclear.

One potential explanation posits that the activation of cytokines and glial cells in the CNS may occur independently of the infiltration of peripheral cytokines and cells. A key factor contributing to this pro-inflammatory response is the excessive production of glucocorticoids during stress. Microglia, which express high levels of glucocorticoid receptors (GR), become primed by elevated levels of glucocorticoids, resulting in a heightened reactivity to subsequent immune challenges after stress exposure [63]. The predominance of the pro-inflammatory effects over the anti-inflammatory properties of glucocorticoids in stressful situations could contribute to the neuroinflammation observed in individuals with MDD. Moreover, studies have shown that neurons regulate microglial activity by secreting signals, including CX3CL1, members of the immunoglobulin superfamily (such as CD200, CD47), neurotrophins, and neurotransmitters [64]. Microglia continuously monitor neuronal activity and adjust their cytokine production and activation status accordingly [65]. Under conditions of chronic stress, disruption of fractalkine signaling, which includes the interaction between neuron-expressed CX3CL1 and microglial CX3CR1, was associated with significant improvements in depressive-like behaviors and cognitive dysfunction compared to controls. Furthermore, a deficiency in CX3CR1 led to alternative microglial activation and decreased production of pro-inflammatory cytokines, ultimately reducing neuroinflammation [66]. Consistently, the levels of CX3CL1 are significantly elevated in patients with MDD, suggesting that communications between neurons and microglia may play a role in neuroinflammation and the progression of depression [67].

Furthermore, research indicates that the administration of labetalol, an adrenergic receptor blocker, significantly attenuated the levels of circulating pro-inflammatory cytokines while having no impact on the expression of IL-1β in the CNS of stressed mice [68]. In animal models of depression, early-life stress significantly increased the expression levels of IL-1β, IL-6, and TNF-α in the brain, while blood levels remained unchanged [69]. Collectively, these findings suggest that different mechanisms may be responsible for the inflammatory processes occurring in the CNS in patients with MDD, separate from the inflammatory mediators entering from the periphery.

In addition, an updated model for the crosstalk between systemic and CNS immunity has been proposed. This model posits that systemic inflammation leads to reduced permeability of the blood–brain barriers, subsequently disrupting solute transport and resulting in reduced brain activity [70]. This disruption may persist even after the resolution of systemic inflammation, impairing brain homeostasis and nutrient transport across the boundaries, ultimately contributing to neuroinflammation and neuronal dysfunctions. Evidence supporting this theory includes observations of increased sickness and volume of the choroid plexus in patients with MDD, reduced perfusion permeability in mouse models of depression, and a significant negative association between plasma CRP levels and the permeability of the blood–brain barrier [71,72,73]. In addition, MRI results support that BBB water permeability is reduced in MDD patients, which may lead to brain metabolic defects [70].

Nonetheless, contradictory evidence has also been reported, showing that the CSF/plasma albumin ratio remained unchanged and did not correlate with elevated peripheral CRP levels in MDD patients, implying that the observed increases in peripheral and central CRP levels in MDD patients may have occurred locally and were not attributed to changes in the BBB permeability [61]. Moreover, one study shows that the expression level of common tight junction proteins, such as claudin-5, is reduced in the hippocampus of patients with MDD [74]. This discrepancy may be due to differences in study populations, methodologies employed, and disease stage, highlighting the complexity of BBB dynamics in the context of MDD.

Recent PET scans have indicated a significant correlation between TSPO expression in the skull and parameningeal areas with both CNS and systemic immune signaling. Aligning with previous reports, despite these associations, CNS neuroinflammation has not demonstrated a direct association with systemic immune markers [75]. This finding highlights the potential functions of the skull and sinuses as intermediate locations between CNS and systemic immune crosstalk in MDD. It raises the hypothesis that immune cells influencing the brain activity may originate from nearby anatomical structures, specifically the skull. Supporting this notion, research has shown that meningeal B cells become activated and exhibit a reduction in number in response to stress, which subsequently triggers the activation of myeloid cells within the meninges, ultimately resulting in behavioral dysregulation [76]. Additionally, the existence of vascular connections among the skull, meninges, and brain parenchyma, coupled with evidence of significant upregulation of skull TSPO expression in conditions such as stroke and neurodegenerative diseases, suggests that inflammation in the skull and meninges plays a crucial role in the interplay between peripheral and neuroinflammation in various pathological conditions [77].

Taken together, the neuroinflammation observed in patients with MDD should not be exclusively linked to the infiltration of systemic inflammatory mediators such as cytokines and immune cells. Activation of the neuroimmune system may result from elevated glucocorticoid levels, changes in neuronal signaling, restricted solute transport across the BBB, and signaling from the skull. Future studies should investigate inflammatory levels in both the peripheral system and the CNS simultaneously, while considering factors such as glucocorticoid levels, neuronal activity, BBB permeability, and the involvement of extra-axial immune cells.

### 2.5. Possible Sources of Systemic Inflammation in MDD Patients

#### 2.5.1. The HPA Axis

**Figure 3:**
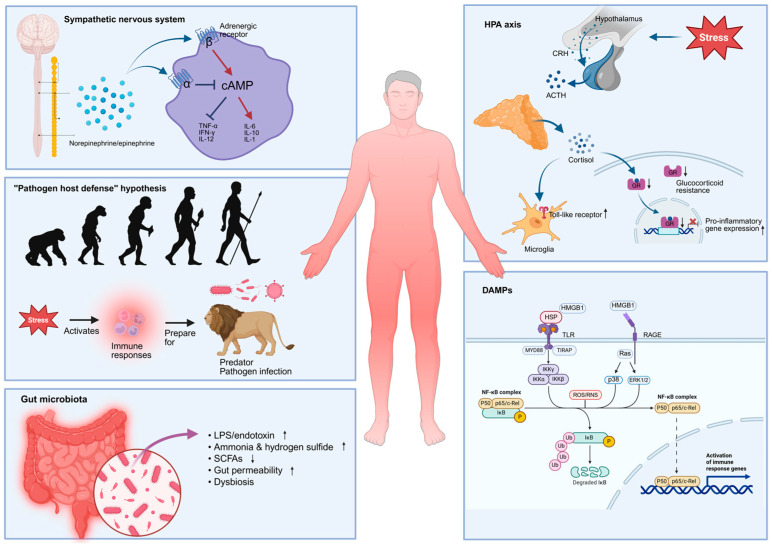
Schematic diagram illustrating potential sources of systemic inflammation in patients with Major Depressive Disorder. Activation of the sympathetic nervous system leads to the release of norepinephrine and epinephrine, which activate adrenergic receptors on immune cells, resulting in the production of both anti-inflammatory and pro-inflammatory cytokines. Cortisol, released from a hyperactive HPA axis, can exert pro-inflammatory effects by upregulating TLR expression on microglia, or by causing glucocorticoid resistance, which hinders cortisol’s immunopressive effects and contributes to inflammation. Throughout evolution, stress indicates potential threats from predators or pathogen infections. Consequently, the immune system is activated in response to stress to prepare individuals for possible injuries and insults. Additionally, dysbiosis of gut microbiota, as well as DAMPs such as HSP, HMGB1, and reactive oxygen/nitrogen species, can further contribute to systemic inflammation. ↑: increase; ↓: decrease.

The HPA axis is a critical neuroendocrine system that is involved in the regulation of stress responses. Its activation is tightly regulated by a negative feedback loop, which prevents excessive activation and overproduction of glucocorticoids. In at least a subset of patients with MDD, there is a consistent observation of hyperactivity in the HPA axis, elevated cortisol levels, and inflammation [78]. Activation of the HPA axis stimulates the synthesis of glucocorticoids from the adrenal gland, which serves as the principal stress hormone and has previously demonstrated anti-inflammatory effects [79]. This gave rise to the hypothesis that activation of the HPA axis plays a role in the inflammation observed in patients with MDD. Figure 3 summarizes potential sources of systemic inflammation in MDD patients.

The theory of “glucocorticoid resistance” in MDD was proposed in the late 1990s, suggesting that glucocorticoid receptors in individuals with MDD demonstrate diminished sensitivity to cortisol and are functionally compromised [80]. This compromised receptor activity disrupts the negative feedback loop, resulting in hyperactivity of the HPA axis. Furthermore, the reduced responsiveness of GRs on immune cells to the immunosuppressive effects of cortisol may lead to an increase in activation of immune cells, ultimately contributing to elevated levels of inflammation in patients with MDD [81].

In addition to the “glucocorticoid resistance” theory, an alternative model has been developed regarding the role of glucocorticoids in inflammation, highlighting their potential pro-inflammatory effects [81]. Previous studies have shown that treatment with GR antagonists reduced pro-inflammatory gene expression in response to stress, indicating that glucocorticoids may contribute to inflammatory processes [82]. Additionally, animal studies have demonstrated that suppression of corticosterone production reduced levels of neuroinflammation and prevented the release of monocytes into the bloodstream during stress [83]. Moreover, glucocorticoids have been found to enhance the expression of receptors on microglia, leading to a primed state that amplifies the inflammatory response to subsequent infections [84]. Collectively, these findings suggest that glucocorticoids may indeed have pro-inflammatory effects under stress, implying that the heightened inflammation observed in patients with MDD could be directly linked to elevated cortisol levels.

#### 2.5.2. The Sympathetic Nervous System (SNS)

The SNS is an important component of the autonomic nervous system. In contrast to the parasympathetic nervous system (PNS), which facilitates the conservation and restoration of body energy, the SNS plays a crucial role in mediating the “fight or flight” response during stress. Moreover, the SNS has been linked to the modulation of immune system functions: upon release, both norepinephrine and epinephrine bind to adrenergic receptors on immune cells and various target organs, modulating the secretion of pro- and anti-inflammatory cytokines, as well as the activation and migration of immune cells [85,86].

Research has shown that stimulating α2-adrenoceptors enhances the release of TNF-α in macrophages following lipopolysaccharide (LPS) stimulation, potentially via the inhibition of intracellular cyclic adenosine monophosphate (cAMP) [87]. Unlike α2-adrenoceptors, activation of β-adrenergic receptors leads to an increase in intracellular cAMP levels. Increase in cAMP then inhibits the synthesis of various pro-inflammatory cytokines such as TNF-α, Interferon gamma (IFN-γ), and Interleukin-12 (IL-12), while facilitating the production of IL-6 and Interleukin-10 (IL-10) [88]. Nonetheless, stimulation of β-adrenergic receptors has also been implicated in the production of IL-1. For example, the β-adrenergic agonist isoproterenol has been demonstrated to promote expression of mRNA for IL-1β in both cultured microglia and human primary monocytes via cAMP-dependent pathways [89,90].

Elevated expressions of pro-inflammatory cytokines are frequently observed in animal models of stress, such as those induced by tailshock. The administration of adrenoceptor antagonists 30 min prior to the exposure of tailshock effectively mitigated the augmentation of IL-1 levels in both plasma and hypothalamus, depending on the specific adrenoceptor targeted [68,91]. Moreover, activation of adrenergic receptors was found to enhance the expression of IL-6 in plasma, as well as IL-1β in both brain tissue and plasma after tailshock [68]. Additionally, the application of noradrenergic reuptake inhibitors markedly intensified the IL-1 response induced by footshock in both the hypothalamus and the spleen [91]. Taken together, these findings suggest that catecholamine signaling plays a substantial role in mediating stress-induced inflammation.

#### 2.5.3. Gut Microbiota

In recent years, the gut–brain axis has gained significant attention across various fields of study, emphasizing the interplay between gut microbiota composition and a range of neurological and psychiatric disorders, including MDD [92]. Our gastrointestinal tract hosts a rich and diverse community of microorganisms that perform essential functions such as metabolism, digestion, immune system regulation, and protection against harmful pathogens [93]. Research has shown a strong association between approximately half of the gut microbiomes and the clinical parameters of MDD patients. Furthermore, studies have demonstrated that probiotics can effectively alleviate depressive symptoms and mitigate inflammation [94].

Gut bacteria possess the ability to metabolize dietary carbohydrates into short-chain fatty acids (SCFAs), which have been demonstrated to exert immunosuppressive and anti-inflammatory effects through the promotion of T cell differentiation and the inhibition of histone deacetylase [95]. Additionally, gut bacteria can degrade proteins through putrefaction, leading to the production of toxic products such as ammonia, hydrogen sulfide, and various amines [96]. Patients with MDD exhibit both increased and decreased abundances of specific bacterial taxa [97]. Notably, genera that are diminished in patients with MDD are known for their capacity to metabolize carbohydrates, whereas those that are increased in these patients are associated with high capacity for protein metabolism. This alteration in microbial composition in MDD patients may contribute to an elevated inflammatory state within the body.

Additionally, a robust positive correlation exists between the microbiota upregulated in MDD patients and the expression of plasma IL-1β [98]. MDD patients exhibit significantly elevated levels of IgM and IgA targeting LPS from various enterobacteria, indicating increased gut permeability and a potential translocation of LPS from the epithelial mucosa into the lamina propria, mesenteric lymph nodes, and systemic circulation. Consequently, this may contribute to systemic inflammatory responses within the body. Indeed, a study involving 41 female MDD participants successfully demonstrated a significant positive correlation between depression and gut permeability, as assessed by the lactulose to mannitol ratio [99]. In animal models, chronic psychological stress has been shown to increase gut epithelial permeability throughout all regions of the gastrointestinal tract. Collectively, an imbalanced microbiota composition and heightened intestinal permeability may contribute to the systemic inflammation observed in patients with MDD.

#### 2.5.4. “Pathogen Host Defense” Hypothesis

From an evolutionary standpoint, depressive symptoms such as social withdrawal, fatigue, and anhedonia serve vital roles in promoting wound healing, combating infections, and enhancing survival. Stress, recognized as a prominent risk factor for depression, holds evolutionary significance by foretelling potential threats such as pathogenic infections and injuries. Consequently, when faced with stress, it is not surprising that the body intensifies immune responses and readies itself for potential health challenges. This evolutionarily ingrained mechanism persists in modern society, despite stressors typically not directly leading to severe infections. Moreover, this evolutionary perspective sheds light on the higher prevalence of depression among females as opposed to males: females generally exhibit a more pronounced and rapid inflammatory reaction compared to males, along with a greater tendency towards depressive behaviors. This gender disparity in depression may serve an evolutionary function by enhancing female resilience against infections and pathogen risks, thereby safeguarding reproductive success in the long term [59].

#### 2.5.5. Damage-Associated Molecular Patterns (DAMPs)

DAMPs are endogenous molecules released from stressed or damaged cells that play crucial roles in the immune response. Given the sterile nature of the systemic inflammation frequently seen in MDD patients, DAMPs may serve as key initiators of these inflammatory responses. Common examples of DAMPs include heat shock proteins (HSP), reactive oxygen and nitrogen species (ROS & RNS), high mobility group box 1 (HMGB1), uric acid, and adenosine triphosphate (ATP). Increased concentrations of DAMPs are frequently observed in response to stress. Upon their release into the extracellular space, DAMPs can activate pattern recognition receptors located on endothelial and various immune cells, thereby initiating both innate and adaptive immune responses. This activation leads to the synthesis of pro-inflammatory cytokines and the subsequent activation of sterile immune responses. For example, HSPs released in response to acute stress are known to bind to the plasma membrane of monocytes, triggering the activation of the NF-κB signaling pathway, resulting in the production of pro-inflammatory cytokines such as TNF-α, IL-1β, and IL-6, as well as nitric oxide [100,101]. Furthermore, excessive production of reactive species in patients with MDD can result in significant damage to RNA, DNA, lipids, and protein functions, generating products exhibiting strong pro-inflammatory properties and neoepitopes [102,103]. In addition, ROS and RNS can directly activate redox-sensitive transcription pathways, leading to the upregulation of pro-inflammatory cytokines. For example, hydrogen peroxide produced by mitochondria is known to activate NF-κB, stimulate inflammasome activity, and enhance the expression of IL-1β [104]. In the CNS, HMGB1 is synthesized in response to stress, which sensitizes microglia and the inflammasome for subsequent inflammatory reactions. Upon its release, HMGB1 interacts with the RAGE receptor and TLR2/4, activating different signaling pathways and facilitating the translocation of NF-κB. This process eventually results in the induction of pro-inflammatory cytokine expression.

## 3. Inflammatory Responses in the Rodent Model of Depression

### 3.1. Animal Studies Confirmed the Bidirectional Relationship Between Inflammation and Depression

Beyond investigating inflammation in individuals with depression, animal models serve as essential tools for elucidating the underlying mechanisms of this mental health condition. Just like in humans, animal studies support a bidirectional relationship between inflammatory responses and depression. Upon exposure to peripheral challenge of endotoxin or pro-inflammatory cytokines, rodents exhibited depression-like behaviors that are sensitive to antidepressant treatment [105]. On the other hand, elevated levels of inflammation in both the periphery and brain have been observed in rodent models of depression, highlighting the role of cytokine signaling in stress-induced depression. For example, the application of immobilization stress in rats significantly increased expression of mRNA for IL-1 in the hypothalamus, accompanied by an increase in biologically active IL-1 levels in the same brain region, while administration of IL-1 receptor antagonist successfully blocked stress-induced depression [106]. The expression level of IL-1β was elevated in the brain and periphery blood after chronic mild stress (CMS), with the mRNA expression level positively correlating to the degree of anhedonia [107]. Both stress and LPS challenges resulted in depressive-like symptoms and elevated expression of pro-inflammatory cytokines in both the brain and serum, alongside increased activation of microglia within the CNS [108].

The role of inflammatory factors in depression was further elucidated with the help of transgenic animal lines, where knock-out or overexpression of specific immune genes significantly altered the manifestation of depression-related symptoms. For example, CMS was associated with a reduction in hippocampal neurogenesis. However, such an effect was not found in IL-1 knock-out mice [109]. Glutaminase is an enzyme that catalyzes the conversion of glutamine to glutamate and is involved not only in glutamate neurotransmission but also in microglia activation and cytokine production [110]. Conditional knockout of glutaminase 1 in microglia effectively mitigated neuroinflammation and depressive-like behaviors both in the peripheral LPS challenge model and chronic restraint model, highlighting the role of microglial glutaminase 1 in neuroinflammation and the development of depression [111,112].

### 3.2. Changes in Brain Lymphatic Function in Animal Models of Depression

The traditional view held that the brain lacked a lymphatic system. However, this view shifted in 2015 when studies first revealed the presence of meningeal lymphatic vessels and demonstrated their ability to maintain brain homeostasis and remove waste products by draining CSF lymphatically through the meningeal lymphatic vessels and the cervical lymphatics [113,114,115]. In the brain, CSF is generated in the choroid plexuses and travels through the ventricles and subarachnoid space. From there, it enters the periarterial space around the cerebral arteries, where it exchanges with interstitial fluid and facilitates waste clearance from the brain parenchyma through the glymphatic system [116]. After interchange, the CSF moves towards the perivenous spaces and eventually drains out of the central nervous system into the deep cervical lymph nodes through the lymphatic vessels [117]. The glymphatic system removes extracellular molecules such as ROS, cytokines, and antigens, and has been shown to be involved in the regulation of disease-associated proteins such as amyloid β and tau [117,118]. Therefore, impairment of the glymphatic system leads to the accumulation of ROS and pro-inflammatory cytokines, triggering the development of neuroinflammation and contributing to psychiatric disorders such as depression.

Brain lymphatic function is influenced by multiple factors, including sleep, aging, physical activity, alcohol consumption, inflammation, and stress levels [119]. In mice subjected to a chronic unpredictable mild stress (CUMS) model of depression, the deep brain lymphatic vessels in the hippocampus and thalamus exhibited a marked decrease in both length and area when compared to the unstressed control group [120]. Moreover, in another study, 8 weeks of CUMS led to depression-like symptoms, microglial activation in the prefrontal cortex, and glymphatic dysfunction. Treatment with polyunsaturated fatty acid supplements significantly reduced depressive-like behaviors and neuroinflammation, all of which were accompanied by restoration of brain glymphatic system function [121]. Similarly, female mice exhibited impairment in meningeal lymphatic function following sub-chronic variable stress, while enhancing the meningeal lymphatics effectively mitigated stress-induced depression-like behaviors and alterations in astrocyte and neuronal function [122]. On the other hand, compromising meningeal lymphatic function notably heightened susceptibility to stress, underscoring the importance of the brain’s lymphatic system in neuroinflammation and stress response [122].

A decline in lymphatic function could lead to a decreased ability for waste clearance and potential toxin accumulation, contributing to the induction of local inflammation. Research has demonstrated that surgical ablation of meningeal lymphatics significantly impairs memory performance, activates microglia, and increases IL-6 levels in the brain [15]. Increased IL-6 expression disrupts the balance between inhibitory and excitatory synapses through both the classical and trans-IL-6 signaling pathways, impairing inhibitory synapse functionality and ultimately contributing to cognitive impairment [15]. Although research on changes in lymphatics among patients with MDD is limited and scarce, exploring alterations in the brain’s lymphatic system and its relationship with neuroinflammation and neuronal dysfunction is an important area that warrants further investigation.

## 4. Conclusions and Future Directions

Taken together, the large number of clinical and pre-clinical studies examined in this review highlight the complex relationship between inflammation and depression. The evidence consistently supports a bidirectional connection, where inflammatory processes can influence depressive symptoms and, conversely, depression may exacerbate inflammation. However, it is essential to recognize that the findings are mixed, indicating a need for further research to reveal the precise nature of this relationship. This review also investigates the distinct yet interconnected aspects of systemic inflammation and neuroinflammation in MDD, emphasizing the potential mechanisms that underlie their interplay and possible explanations for observed desynchrony between systemic and neuroinflammation. Identified sources of systemic inflammation include hyperactivity of the HPA axis, dysregulation of the sympathetic nervous system, gut microbiota imbalance, the “pathogen host defense” hypothesis, and DAMPs. These factors not only contribute to systemic inflammation but may also exacerbate neuroinflammatory processes. The insights gained from both clinical studies and animal models emphasize the need for a multifaceted approach to understanding the role of inflammation in MDD. Future research involving patients with MDD should simultaneously investigate systemic inflammation and neuroinflammation through PET imaging, CSF, and blood analyses. It is crucial to consider the symptom heterogeneity among MDD patients and the alterations in the brain lymphatic system, correlating these findings with specific dimensions of depression. Moreover, longitudinal studies are needed to link inflammatory profiles with changes in depression symptoms. Overall, this review underscores the importance of ongoing research in this field to deepen our understanding of the complex relationships between inflammation and depression, ultimately facilitating the development of more effective prevention and treatment approaches for MDD.

## Figures and Tables

**Table 3:** Correlation between peripheral blood inflammatory markers and neuroinflammation markers in MDD patients.

Author	Peripheral Blood Inflammatory Marker	Neuroinflammation Marker	Correlation
Hannestad et al. [34]	hsCRP	TSPO binding	No
Holmes et al. [33]	IFN-γ, TNF-α, IL-6, IL-8, IL-1β and CRP	TSPO binding	No
Lindqvist et al. [31]	IL-6, IL-1β, IL-8, and TNF-α	IL-6, IL-1β, IL-8, and TNF-α in CSF	No
Sasayama et al. [44]	IL-6	IL-6 in CSF	No
Setiawan et al. [42]	IL-1β, IL-6, TNF-α, and CRP	TSPO binding	No
Hestad et al. [45]	Eotaxin, IP-10, MIP-1β	Eotaxin, IP-10, MIP-1β in CSF	High correlation (>0.4)
Levine et al. [60]	TNF	IL-1β in CSF	Significant positive correlation
Felger et al. [61]	CRP	CRP in CSF	Strong correlation (r = 0.855, *p* < 0.001)
Schubert et al. [62]	CRP	TSPO binding	No

## Data Availability

Not applicable.

## Abbreviations

The following abbreviations are used in this manuscript:

ATP	Adenosine triphosphate
AP	Area postrema
BBB	Blood–brain barrier
cAMP	Cyclic adenosine monophosphate
CCL2	C-C motif chemokine ligand 2
CD4	Cluster of differentiation 4
CD19	Cluster of differentiation 19
CD14	Cluster of differentiation 14
CD16	Cluster of differentiation 16
CD25	Cluster of differentiation 25
CD30	Cluster of differentiation 30
CD47	Cluster of differentiation 47
CD200	CD200 molecule
CNPase	2′,3′-cyclic-nucleotide 3′-phosphodiesterase
CMS	Chronic mild stress
CNS	Central nervous system
COX2	cycloocygenase-2
CP	Choroid plexus
CRP	C-reactive protein
CSF	Cerebrospinal fluid
CUMS	Chronic unpredictable mild stress
CX3CL1	C-X3-C motif chemokine ligand 1
CX3CR1	C-X3-C motif chemokine receptor 1
DAMP	Damage-associated molecular pattern
Foxp3	Forkhead box protein P3
GFAP	Glial fibrillary acidic protein
GR	Glucocorticoid receptor
HLA-DR	Human leukocyte antigen, DR isotypes
HMGB1	High mobility group box 1
HPA	Hypothalamic–pituitary–adrenal
hs-CRP	high-sensitivity C-reactive protein
HSP	Heat shock protein
IBA1	Ionized calcium-binding adapter molecule 1
IFN-α	Interferon alfa
IFN-γ	Interferon gamma
IL-1α	Interleukin-1α
IL-1β	Interleukin-1β
IL-1RA	Interleukin 1 receptor antagonist
IL-2	Interleukin-2
IL-4	Interleukin-4
IL-5	Interleukin-5
IL-6	Interleukin-6
IL-7	Interleukin-7
IL-8	Interleukin-8
IL-10	Interleukin-10
IL-12	Interleukin-12
IL-13	Interleukin-13
IL-17	Interleukin-17
IL-18	Interleukin-18
IL-21	Interleukin-21
IL-23	Interleukin-23
IL-33	Interleukin-33
IL-35	Interleukin-35
IP-10	Interferon-inducible protein 10
LPS	Lipopolysaccharide
MCP-1	Monocyte chemoattractant protein-1
MDD	Major depressive disorder
MIP-1β	macrophage inflammatory protein 1 beta
MRI	Magnetic resonance imaging
NF-κB	Nuclear factor kappa B
Olig2	Oligodendrocyte transcription factor 2
PET	Positron emission tomography
PNS	Parasympathetic nervous system
RNS	Reactive nitrogen species
ROS	Reactive oxygen species
RAGE	Receptor for advanced glycation end products
SCFA	Short-chain fatty acids
SFO	Subfornical organ
SNS	Sympathetic nervous system
SOCS1	Suppressor of cytokine signaling 1
SOCS2	Suppressor of cytokine signaling 2
SOCS3	Suppressor of cytokine signaling 3
SSRI	Selective serotonin reuptake inhibitor
Th1/Th2	T helper type 1 (Th1) and T helper type 2 (Th2)
Th17	T helper 17 cell
TLR	Toll-like receptor
TNF-α	Tumor necrosis factor-alpha
TSPO	Translocator protein
VOLT	Vascular organ of lamina terminalis
